# Expanded HIV Clinic–Based Mental Health Care Services: Association With Viral Suppression

**DOI:** 10.1093/ofid/ofz146

**Published:** 2019-03-18

**Authors:** Raina Aggarwal, Michael Pham, Rebecca Dillingham, Kathleen A McManus

**Affiliations:** 1 School of Medicine, University of Virginia, Charlottesville, Virginia; 2 Division of Infectious Diseases and International Health, University of Virginia, Charlottesville, Virginia; 3 Center for Health Policy, University of Virginia, Charlottesville, Virginia

**Keywords:** engagement in care, HIV, mental health care, substance use disorder, viral suppression

## Abstract

**Background:**

An academic Ryan White HIV/AIDS Program clinic increased co-located mental health care (MH) services in 2013. The study objectives were to characterize the changing demographics of the people living with HIV (PLWH) who initiated MH and to determine MH initiation’s association with HIV outcomes.

**Methods:**

The cohort included PLWH who received clinic-based MH services from 2012 to 2014. Cohorts A and B initiated MH before or during 2012 and during 2013–2014, respectively. Demographics were compared for the 2 cohorts, and for Cohort B, pre/post–MH initiation clinical outcomes were compared.

**Results:**

Compared with Cohort A (n = 130), Cohort B (n = 181) had 3 times the participants with CD4 counts <200 (*P* = .02). One-third of Cohort B had detectable viral loads compared with <20% of Cohort A (*P* = .01). Cohort B received more substance use diagnoses (*P* = .005). Pre/post–MH initiation, engagement in care did not change. For Cohort B, MH initiation was associated with increased rates of viral suppression. For those who were prescribed antiretroviral therapy more than 1 year before MH initiation, participants who were older and nonblack were more likely to achieve viral suppression.

**Conclusions:**

PLWH who gained access to MH in 2013–2014 were more likely to have lower CD4 counts and detectable viral loads. Engagement in care did not increase with initiation of MH, but initiation of MH was associated with higher rates of viral suppression. Younger and minority patients may not have benefited as much from increased access to co-located MH and substance use services.

The United States’ HIV viral suppression rate has not met the National HIV/AIDS Strategy and Joint United Nations Program on HIV/AIDS’ goal of having 90% of people living with HIV (PLWH) achieve viral suppression [1, 2]. The Centers for Disease Control and Prevention estimates that approximately 36% of PLWH in the United States are aware of their HIV diagnosis but are currently not virally suppressed [3]. Optimizing the HIV care and engagement of PLWH should include addressing mental health and substance use issues. PLWH with mental health diagnoses and substance use issues have been found to be less engaged in HIV care [4], have decreased medication adherence [5], achieve viral suppression at lower rates [6–9], and experience higher rates of morbidity and mortality [5, 10].

Given these issues associated with mental health diagnoses and an unmet need for MH services, an academic Ryan White HIV/AIDS Program (RWHAP) clinic increased co-located clinic-based mental health care (MH) services including psychiatry, psychology, and substance use counseling in 2013. Evaluating this change provided a unique opportunity to assess the effects of MH service expansion on HIV outcomes. The aims of this study were to characterize the changing demographics of PLWH who initiated MH and to determine the association of MH initiation with HIV outcomes, specifically engagement in HIV care and HIV viral suppression. We hypothesized that PLWH who received MH services in 2012 would differ in demographics (older age, higher CD4 counts, higher rates of viral suppression, and fewer issues with substance use) than those who received MH services in 2013 and 2014. We also hypothesized that PLWH who started receiving MH services in 2013 and 2014 would have higher rates of engagement in HIV care and viral suppression in the year after their initial MH visit compared with the year before establishing MH. This hypothesis is based on the theory that if clients’ mental health and substance use issues are addressed and managed by an MH provider, the client may be able to better engage in HIV care, take daily medication, and achieve viral suppression. This study sought to provide new information about the role of co-located MH in achieving optimal clinical outcomes for PLWH with the goal of informing policies such as encouraging increased support and resources for co-located mental health and HIV care. The results could also possibly catalyze future insurance benefit modifications to include more robust coverage for mental health and substance use disorders.

## METHODS

### Expansion of MH Services

Before and during 2012, the University of Virginia’s RWHAP HIV Clinic had 1 grant-supported psychiatrist who provided MH services embedded in the same clinic location as the RWHAP HIV medical visits. Additionally, there were intermittently psychiatry residents who were available for MH services as well. These MH services were in high demand, and all open appointments were booked. To increase MH services to fulfill an unmet need, the studied RWHAP clinic requested increased RWHAP resources for mental health, and, with increased funding, the clinic was able to hire Clinical Psychologists and a Certified Substance Abuse Counselor. They provide services embedded in the medical clinic in separate consulting rooms that are equipped with comfortable seating and no medical devices.

MH referrals are placed by medical providers and can also be triggered by other staff of the RWHAP clinic. MH providers focus on any referral or patient concern. Initial appointments are 1 hour. Follow-up appointments are 30 minutes. When possible, a warm hand-off is used to introduce the patient to the MH provider. 

In 2012, 19% of all HIV Clinic clients (n = 672) were receiving MH services at the HIV Clinic. By 2014, 41% of all clinic clients (n = 753) had at least 1 MH visit at the HIV Clinic.

### Cohort

This retrospective cohort study included PLWH who received HIV Clinic–based MH services at the studied RWHAP HIV Clinic from January 1, 2012, through December 31, 2014. Receipt of MH services was defined as at least 1 visit with an MH provider, including a psychiatrist, psychologist, or substance use counselor. Cohort A initiated MH before or during 2012, and Cohort B initiated MH after the MH expansion, during 2013 or 2014.

### Analysis

The total number of MH visits and the number of visits with different types of MH providers were tracked annually from 2012 to 2014. Descriptive statistics were used to assess MH services over time.

### Demographics

Data at the time of first MH visit were collected, including age, race/ethnicity (black, Hispanic, white, other), self-reported gender (cis-gender male, cis-gender female, transgender), financial status (in terms of percentage of the Federal Poverty Level), CD4 cell count, HIV viral load, time from HIV diagnosis to MH initiation, time from linkage to HIV medical care to MH initiation, antiretroviral therapy (ART) initiation, and time from ART initiation to first MH visit. These data were compared between Cohort A and Cohort B using *t* tests for continuous data and chi-square tests for categorical data. Additionally, all mental health diagnoses that a participant received in the year after MH initiation were collected. Diagnoses were determined using coding by the International Classification of Diseases, Ninth Revision (ICD-9), and were then grouped into categories: psychotic conditions, neurotic/anxiety disorders, personality disorders, sexual disorders, substance use disorders, acute stress reaction/adjustment disorders, and depressive disorders. The distribution of mental health diagnoses across the categories was compared between Cohorts A and B using the chi-square test. All analyses in this study were performed with a significance level of .05 using IBM SPSS Statistics software, version 24, and R.

### Engagement in HIV Care

The engagement in HIV care of participants in the year before and the year after MH initiation were compared for Cohort B members who had established care at least 12 months before MH initiation. Engagement in HIV care was assessed using the Health Resources and Services Administration’s (HRSA’s) HIV/AIDS Bureau Performance Measures’ measurement for medical visit frequency, defined as at least 1 medical visit in each 6-month period with at least 60 days between visits [11]. McNemar’s test was used to compare the proportion of participants who met the medical visit frequency quality metric in the year before and the year after initiating MH.

### Viral Suppression

Viral suppression was also analyzed. Viral suppression was defined as an undetectable viral load or <200 RNA copies per mL, and participants were assessed for a good viral outcome, defined as maintaining or achieving viral suppression [12]. If a participant had more than 1 viral load in a year, the first viral load within the year before MH initiation and the last viral load within the year after MH initiation were used. For Cohort B, viral suppression rates were calculated during the year before and the year after MH initiation. McNemar’s test was used to compare the proportion of participants with a good viral outcome in the year before and after MH initiation. McNemar’s test was repeated as a sensitivity analysis, assuming that any missing viral loads were detectable to offer a worst case scenario for the viral suppression outcome.

As an exploratory analysis to assess if this outcome was different based on the timing of ART initiation, we looked at groups within Cohort B. Cohort B1 was prescribed ART at least 12 months before MH initiation, Cohort B2 was prescribed ART at least 1 day to 364 days before MH initiation, and Cohort B3 was prescribed ART on the same day as MH initiation or after MH initiation. McNemar’s test and the sensitivity analysis were repeated for B1 and B2.

For participants in Cohort B who had initiated ART at least 12 months before their MH initiation, binary logistic regression was used to assess the strength and significance of the association between a good viral outcome after initiating MH and key variables. The variables assessed include age, race/ethnicity (black compared with nonblack), gender (cis-gender male compared with non-cis-gender male), financial status (<100% FPL compared with >100% FPL), pre-MH viral suppression, time since HIV diagnosis, and time since linkage to HIV care at the studied HIV Clinic.

## RESULTS

A total of 311 participants met inclusion criteria by having at least 1 HIV Clinic–based MH service at the studied RWHAP HIV Clinic from January 1, 2012, through December 31, 2014. Cohort A included 130 participants who initiated MH before or during 2012, and Cohort B included 181 participants who initiated MH after the MH expansion, during 2013 or 2014.

**Figure 1.**
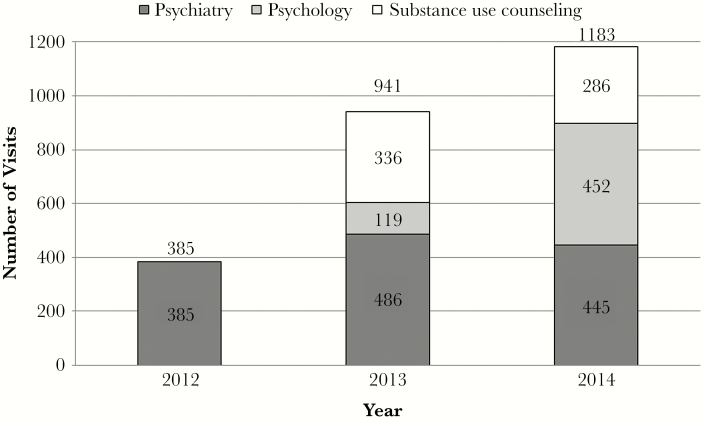
In 2012, there were 385 co-located mental health care visits at the studied Ryan White HIV/AIDS Program clinic, and all 385 visits were with psychiatrists. In 2013, 486 visits or 52% were with psychiatrists, 336 or 36% were with substance use counselors, and 119 or 13% were with psychologists. In 2014, 445 visits or 38% were with psychiatrists, 452 or 38% were with psychologists, and 286 or 24% were with substance use counselors.

MH visits increased from 385 in 2012 to 941 in 2013 and 1183 in 2014. In 2012, all 385 visits were with psychiatrists. In 2013, 486 visits or 52% were with psychiatrists, 336 or 36% were with substance use counselors, and 119 or 13% were with psychologists. In 2014, 445 visits or 38% were with psychiatrists, 452 or 38% were with psychologists, and 286 or 24% were with substance use counselors (Figure 1). Psychology visits increased 380% from 2013 to 2014.

### Demographics

Significant differences at the time of MH initiation were seen between Cohort A and Cohort B for age, financial status, CD4 count, and viral load status (Table 1). Cohort B was younger at the time of MH initiation than Cohort A; Cohort B had a mean age of 42 years, whereas Cohort A had a mean age of 46 years (*P* < .001). Distribution of financial status was not similar between the 2 cohorts. Cohort B had more participants ≤200% FPL (*P* = .05). Compared with the percentage of Cohort A with CD4 cell counts <200, Cohort B’s percentage of participants was almost 3 times as high (*P* = .02). About one-third of Cohort B had detectable viral loads, compared with <20% of Cohort A (*P* = .003). There were no significant differences in demographics between Cohort A and Cohort B for race/ethnicity and gender.

**Table 1.** Characteristics of Cohorts

Characteristic	All, No. (%)	Cohort A, No. (%)	Cohort B, No. (%)	*P* Value
All	311	130	181	
Age, mean, y	43.9	45.9	42.4	<.001
Race/ethnicity				.5
Black	125 (40.2)	50 (38.5)	75 (42.0)	
Hispanic	18 (5.8)	6 (4.6)	12 (6.6)	
White	166 (53.4)	74 (56.9)	92 (50.3)	
Other	2 (0.6)	0 (0.0)	2 (1.1)	
Gender				.2
Male	211 (67.8)	90 (69.2)	121 (66.9)	
Female	95 (30.5)	36 (27.7)	59 (32.6)	
Transgender	5 (1.6)	4 (3.1)	1 (0.6)	
Financial status				.05
>400% FPL	7 (2.3)	4 (3.1)	3 (1.7)	
301%–400% FPL	7 (2.3)	0 (0.0)	7 (3.9)	
201%–300% FPL	30 (9.6)	16 (12.3)	14 (7.7)	
101%–200% FPL	69 (22.2)	24 (18.5)	45 (24.9)	
<100% FPL	198 (63.7)	86 (66.2)	112 (61.9)	
CD4 cell count^a^				.02
>500	149 (54.0)	61 (62.9)	88 (49.2)	
201–500	96 (34.8)	31 (32.0)	65 (36.3)	
≤200	31 (11.2)	5 (5.2)	26 (14.5)	
Viral load^b^				.003
Detectable	80 (25.8)	22 (16.9)	58 (32.0)	
Undetectable	230 (74.2)	108 (83.1)	123 (68.0)	
Time since HIV diagnosis, mean, y	10.0	10.9	9.4	.09
Time from HIV care initiation to MH initiation, mean, y	4.9	5.1	4.6	.2
ART initiated before MH initiation	288 (93.5)	124 (95.4)	164 (90.6)	.1
Time from ART initiation to MH visit^c^				
At least 12 mo before	230 (74.7)	113 (88.3)	117 (65.0)	<.001
0–12 mo before	45 (14.6)	10 (7.8)	35 (19.4)	
After MH	33 (10.7)	5 (3.9)	28 (15.6)	
Mental health diagnoses^d^				.005
Psychotic conditions	156 (28.4)	96 (34.5)	60 (22.1)	
Neurotic/anxiety disorders	87 (15.8)	48 (17.3)	39 (14.4)	
Personality disorders	13 (2.4)	5 (1.8)	8 (3.0)	
Sexual disorders	5 (0.9)	1 (0.4)	4 (1.5)	
Substance use disorder	152 (27.7)	67 (24.1)	85 (31.4)	
Acute stress reaction/adjustment disorder	37 (6.7)	11 (4.0)	26 (9.6)	
Depressive disorder	99 (18.0)	50 (18.0)	49 (18.1)	

Abbreviations: ART, antiretroviral therapy; FPL, Federal Poverty Level; MH, mental health care.

^a^Cohort A: 97 participants had a CD4 cell count available; Cohort B: 179 participants had a CD4 cell count available.

^b^HIV viral load quantified in copies/mL.

^c^Cohort A: 128 participants were prescribed ART by the end of the study period; Cohort B: 180 participants were prescribed ART by the end of the study period.

^d^Each participant could have multiple mental health diagnoses. Cohort A: 278 diagnoses were identified; Cohort B: 271 diagnoses were identified. Diagnoses were determined using coding from the International Classification of Diseases, Ninth Revision (ICD-9). ICD-9 codes 290–299 were used to identify subjects with psychotic conditions, 300 to identify neurotic/anxiety disorders, 301 to identify personality disorders, 302 to identify sexual disorders, 303–305 to identify substance use disorders, 308–309 to identify acute stress reaction/adjustment disorders, and 311 to identify depressive disorders.

There was no difference between the cohorts in terms of average time since HIV diagnosis, time since initiation of HIV medical care at the studied HIV Clinic, or percent prescribed ART before MH initiation. There were differences between Cohort A and Cohort B in terms of time from ART start to MH initiation (Table 1). Eighty-eight percent of Cohort A was prescribed ART at least 1 year before their first MH visit, compared with 65% of Cohort B. Ten percent of Cohort A was prescribed ART between 1 and 365 days before their first MH visit, compared with 35% of Cohort B. Less than 5% of Cohort A was prescribed ART after their first MH visit, compared with 16% of Cohort B. More members of Cohort B were prescribed ART between 1 and 364 days before MH initiation or after MH initiation (*P* < .001).

There were also differences in the types of MH diagnoses made in the year after MH initiation (Table 1). In the year after MH initiation, participants in Cohort B received more substance use diagnoses and more acute stress reaction/adjustment disorder diagnoses compared with Cohort A (*P* = .005).

### Engagement in HIV Care

For participants in Cohort B who had established HIV care at least 1 year before establishing MH (n = 122), there was not a significant difference between meeting the HRSA quality metric for HIV medical visit frequency before and after MH initiation. Five percent of participants did not meet the quality metric before or after MH initiation. Eighteen percent of participants only met the quality metric before MH initiation, and 11% of participants only met the metric after MH initiation. Sixty-six percent of participants met the quality metric in the year before initiating MH and the year after MH initiation (*P* = .2).

### Viral Suppression

**Figure 2.**
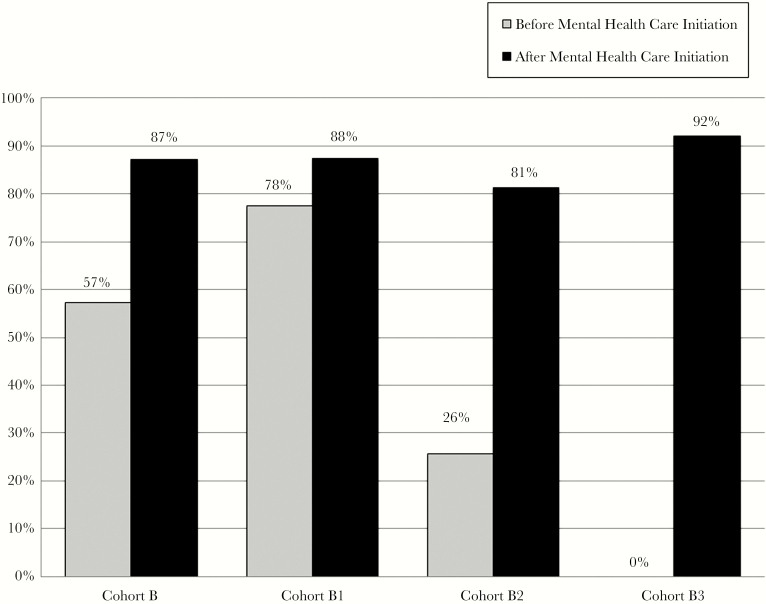
This figure demonstrates the viral suppression rates before and after mental health care (MH) initiation for Cohort B and subgroups based on a complete case analysis. Overall, for Cohort B participants (n = 170), viral suppression was 57% in the year before MH initiation and 88% in the year after MH initiation (*P* < .001). For Cohort B1 participants (n = 113), 78% were virally suppressed before initiation of MH, and 88% achieved or maintained viral suppression (*P* = .02). For Cohort B2 participants (n = 32), 26% were virally suppressed before initiation of MH, and 81% achieved or maintained viral suppression after MH initiation (*P* < .001). For Cohort B3 participants (n = 26), 0% were detectable before initiation of MH as they had not yet been prescribed antiretroviral therapy, and 93% achieved viral suppression after MH initiation.

Overall for Cohort B participants with viral load data available before and after MH initiation (n = 170), viral suppression was 57% in the year before MH initiation and 88% in the year after MH initiation (*P* < .001). Figure 2 demonstrates the viral suppression rates before and after MH initiation based on a complete case analysis for Cohort B and all subgroups. In a sensitivity analysis with the assumption that all missing viral loads are detectable, the viral suppression rate was 57% in the year before MH initiation and 82% in the year after MH initiation (*P* < .001). One participant was an elite controller and had not started ART at the end of the study period. They were not included in these analyses.

For the exploratory analysis, which included Cohort B1 participants who were prescribed ART at least 1 year before MH initiation (n = 120), time from ART prescription to MH initiation was 5.3 years on average, with a median of 5.0 years and a range of 1 to 13 years. For Cohort B1 participants with viral load data available before and after MH initiation (n = 113), 12% were not virally suppressed after initiation of MH, and 88% achieved or maintained viral suppression (*P* = .02). In a sensitivity analysis with the assumption that all missing viral loads are detectable, 17% would have been detectable after initiation of MH and 83% would have achieved or maintained viral suppression (*P* = .2).

For Cohort B2 participants who were prescribed ART within 1 year of MH initiation (n = 35), time from ART prescription to MH initiation was 132 days on average, with a median of 107 days and a range of 31 to 372 days. For Cohort B2 participants with viral load data available before and after MH initiation (n = 32), 19% were not virally suppressed after initiation of MH, and 81% achieved or maintained viral suppression (*P* < .001). In a sensitivity analysis with the assumption that all missing viral loads are detectable, 26% would have been detectable after initiation of MH and 74% would have achieved or maintained viral suppression (*P* < .001).

For Cohort B3 participants (n = 26), who were prescribed ART at the time of or after MH initiation, time from MH initiation to ART prescription was 67 days on average, with a median of 17 days and a range of 0 to 114 days. For Cohort B3 participants with viral load data available after MH initiation (n = 26), 8% were detectable after initiation of MH, and 93% achieved viral suppression. With the assumption that all missing viral loads are detectable, 11% would have had a detectable viral load after initiation of MH, and 89% would have achieved viral suppression.

For participants in Cohort B who had initiated ART at least 12 months before their MH initiation, each additional year increase in age was associated with a higher likelihood of a good viral outcome (odds ratio [OR], 1.06; 95% confidence interval [CI], 1.00–1.13; *P* = .04). When comparing black race/ethnicity with other race/ethnicity groups, black participants were less likely to achieve a good viral outcome (OR, 0.30; 95% CI, 0.09–0.95; *P* = .04). If a participant had achieved viral suppression before MH initiation, they were more likely to have a good viral outcome (OR, 13.67; 95% CI, 3.79–49.33; *P* < .001). Gender (cis-gender male compared with non-cis-gender male), financial status (≤100% FPL compared with >100% FPL), time since HIV diagnosis, and time since linkage to HIV care at the studied HIV Clinic were each individually not associated with viral suppression in univariate logistic regression.

## DISCUSSION

Over the course of 2 years from 2012 to 2014, the studied RWHAP clinic more than doubled the percentage of clinic clients who were engaged in MH. This was mostly achieved through increased availability of MH with the addition of psychologists and a substance use counselor. This MH expansion was supported by RWHAP grants. The demographic data indicate that PLWH who gained access to expanded MH services in 2013–2014 were younger, had lower incomes, and had lower CD4 counts at the time of MH initiation. Although there was not a significant difference between the cohorts in terms of time since diagnosis or time since linkage to care, Cohort B was more likely to not yet be prescribed ART and to have a detectable viral load.

With the MH expansion, participants received more substance use diagnoses. This could have been increased awareness and identification of these issues by medical providers once the resources were available to address substance use issues in the clinic. PLWH with substance use experience higher rates of mortality and morbidity [10]. Given the worsening of the substance use disorder epidemics, increasing awareness and treatment of substance use issues in RWHAP clinics is critical for the health of PLWH and to curb any more injection drug use–related HIV outbreaks [13, 14].

Our study did not demonstrate our hypothesized improvement in clients’ engagement in HIV care after MH initiation compared with their pre–MH initiation engagement. Two-thirds of clients maintained engagement in HIV care before and after MH initiation. This is in contrast to a recent paper that demonstrated improved retention in care with more than 3 MH visits for PLWH who were newly initiating care and received a bundled intervention to improve retention in care [15]. Our study population differed, as the clients were not all newly initiating HIV medical care. It is possible that the different findings relate to the different populations or to a difference between initiating MH and increased MH utilization. In our study, the rates of engagement in HIV care were 84% in the pre-MH year and 77% for the post-MH year. When averaged, these are equal to the national average of retention in care for RWHAP clients in 2014 [16]. This decline in engagement in HIV care is in the opposite direction of the viral suppression changes that Cohort B demonstrated. This supports that the HIV care cascade is not a strict stepwise progression, and frameworks that incorporate care transitions may improve our understanding of the HIV health care delivery system’s gaps [17].

Importantly, initiation of MH services was associated with increased rates of viral suppression for this population overall and for the complete case analysis and the sensitivity analysis demonstrating the worst case scenario. In exploratory analyses, it appears that this effect may have been driven by clients who initiated ART between 1 and 364 days before MH initiation and by clients who initiated ART after MH initiation. Additionally, for those who started ART at the time of MH initiation or after, approximately 90% were virally suppressed within 1 year after MH initiation. This group meets the United States’ National HIV/AIDS Strategy and Joint United Nations Program on HIV/AIDS’ goal of 90% of PLWH achieving viral suppression [1, 2]. Given the lack of significant differences in measures of engagement in HIV care in the year before and the year after MH initiation, the data do not suggest that the mechanism for this increase in viral suppression is increased engagement in HIV care. Given that we were comparing individuals with themselves in a pre/post-MH analysis, it is possible that the increased access to co-located MH and substance use services contributed to high-risk PLWH achieving optimal HIV outcomes. This has major implications, as viral suppression translates to less HIV transmission and fewer new infections [18–20]. Lack of access to MH services among PLWH could be contributing to disparities in medical outcomes.

Although the numbers in our study are small, the binary logistic regression raises some concerning issues regarding disparities in improvement in outcomes for age and race/ethnicity. Our findings demonstrate that for clients who had been prescribed ART for over a year before MH initiation, younger clients and black clients were less likely to achieve viral suppression after MH initiation. This raises questions about the way that we deliver MH services to these clients and whether we need to reconsider how to best support them. Increased age being associated with a higher likelihood of viral suppression is consistent with national data that PLWH over the age of 50 do achieve higher rates of viral suppression than younger PLWH [21]. A review of 70 qualitative studies on developing and improving interventions for black men who have sex with men (MSM) with HIV or who are at risk for HIV recommends that mental health interventions should be combined with structural-level interventions to increase access to stable housing and employment [22]. Future work to explore this could include a qualitative study with younger and/or black clinic clients to discuss what changes to MH services or what additional services would be most helpful for them.

The limitations of this work include that it is a relatively small study that examined an expansion of clinic resources. This retrospective study examined associations, and causality cannot be inferred. Moreover, the study did not compare those who initiated MH with those who did not. Future work should compare viral suppression rates between those who established MH and those who did not in an effort to assess the causal nature of the relationship between MH initiation and viral suppression. Additionally, we did not study the impact of the number of MH visits or the impact of adherence with recommended MH appointments. These studies would require a more extensive chart review of sensitive psychiatry notes and could be performed in the future. Given that the study focused on co-located MH services, it is possible that there were additional clients who received off-site MH and were not included in the study.

More RWHAP clinics should pursue RWHAP support to start or expand co-located HIV medical care and MH services. It is an opportunity to improve viral suppression outcomes for vulnerable populations, and this is pressing given the worsening of the opioid and substance use crises. Moreover, in states with expanded Medicaid or robust AIDS Drug Assistance Program–funded Qualified Health Plan purchasing, RWHAP clinics should consider partnering with MH providers to offer co-located services. This would be another possible way to leverage expanded coverage of MH to improve outcomes for PLWH.
